# Differences in the cost and environmental impact between the current diet in Brazil and healthy and sustainable diets: a modeling study

**DOI:** 10.1186/s12937-024-00973-x

**Published:** 2024-07-09

**Authors:** Thaís Cristina Marquezine Caldeira, Stefanie Vandevijvere, Boyd Swinburn, Sally Mackay, Rafael Moreira Claro

**Affiliations:** 1 Postgraduate Program in Public Health, Medical School, Federal University of Minas Gerais, Avenue Professor Alfredo Balena, 190, Santa Efigênia, Belo Horizonte 30130-100 Brazil; 2 Sciensano, Brussels, Belgium; 3 Faculty of Medical and Health Sciences, Epidemiology and Biostatistics, University of Auckland, Auckland, New Zealand; 4 Nutrition Department, Federal University of Minas Gerais, Belo Horizonte, Brazil

**Keywords:** Diet, Cost, Sustainable development, Public health, EAT-Lancet diet, Brazil

## Abstract

**Background:**

While healthy and sustainable diets benefit human and planetary health, their monetary cost has a direct impact on consumer food choices. This study aimed to identify the cost and environmental impact of the current Brazilian diet (CBD) and compare it with healthy and sustainable diets.

**Methods:**

Data from the Brazilian Household Budget Survey 2017/18 and the Footprints of Foods and Culinary Preparations Consumed in Brazil database were used for a modeling study comparing the cost of healthy and sustainable diets (based on the Brazilian Dietary Guidelines (BDG) diet and the EAT-Lancet diet) versus the CBD. The DIETCOST program generated multiple food baskets for each scenario (Montecarlo simulations). Nutritional quality, cost, and environmental impact measures (carbon footprint (CF) and water footprint (WF)) were estimated for all diets and compared by ANOVA. Simple linear regressions used standardized environmental impacts measures to estimate differentials in costs and environmental impacts among diets scenarios.

**Results:**

We observed significant differences in costs/1000 kcal. The BDG diet was cheaper (BRL$4.9 (95%IC:4.8;4.9) ≈ USD$1.5) than the CBD (BRL$5.6 (95%IC:5.6;5.7) ≈ USD$1.8) and the EAT-Lancet diet (BRL$6.1 (95%IC:6.0;6.1) ≈ USD$1.9). Ultra-processed foods (UPF) and red meat contributed the most to the CBD cost/1000 kcal, while fruits and vegetables made the lowest contribution to CBD. Red meat, sugary drinks, and UPF were the main contributors to the environmental impacts of the CBD. The environmental impact/1000 kcal of the CBD was nearly double (CF:3.1 kg(95%IC: 3.0;3.1); WF:2,705 L 95%IC:2,671;2,739)) the cost of the BDG diet (CF:1.4 kg (95%IC:1.4;1.4); WF:1,542 L (95%IC:1,524;1,561)) and EAT-Lancet diet (CF:1.1 kg (95%IC:1.0;1.1); WF:1,448 L (95%IC:1,428;1,469)). A one standard deviation increase in standardized CF corresponded to an increase of BRL$0.48 in the cost of the CBD, similar to standardized WF (BRL$0.56). A similar relationship between the environmental impact and the cost of the BDG (CF: BRL$0.20; WF: BRL$0.33) and EAT-Lancet (CF: BRL$0.04; WF: BRL$0.18) was found, but with a less pronounced effect.

**Conclusions:**

The BDG diet was cost-effective, while the EAT-Lancet diet was slightly pricier than the CBD. The CBD presented almost double the CF and WF compared to the BDG and EAT-Lancet diets. The lower cost in each diet was associated with lower environmental impact, particularly for the BDG and EAT-Lancet diets. Multisectoral public policies must be applied to guide individuals and societies towards healthier and more sustainable eating patterns.

**Supplementary Information:**

The online version contains supplementary material available at 10.1186/s12937-024-00973-x.

## Background

Food systems and climate changes are strongly related [1, 2]. Globally, food production contributes significantly (approximately 20 to 35%) to greenhouse gas (GHG) emissions, deforestation, land use change, biodiversity loss, and water pollution [1, 3]. This impact has been aggravated in recent decades due to changes in population dietary patterns, resulting from a reduction in the consumption of natural and minimally processed foods and an increase in the consumption of ultra-processed foods (UPF) and animal-source foods, especially in low- and middle-income countries (LMICs) [4].

Most evidence points to the benefits of a plant-based diet, with moderation in the consumption of animal foods, refined grains, sugars, and UPFs, for human and planetary health [2]. Based on this evidence, the EAT-Lancet Commission developed an EAT-Lancet Planetary Diet, which sets out global goals for food systems that favor a healthy and sustainable diet [5]. The promotion of healthy and sustainable diets has also entered the agenda of different countries, with approximately 37 countries having sustainability principles mentioned in their food-based dietary guidelines [6], such as principles about the consumption of plant-based and animal-based foods, environmental impacts, biodiversity, food packaging and food waste [2, 3, 6, 7]. In Brazil, the Dietary Guidelines for the Brazilian population, published by the Ministry of Health in 2014, emphasize the importance of sustainability in food consumption [8]. The Brazilian Dietary Guidelines (BDG) include among its dietary principles: ​​always prefer natural or minimally processed foods to UPF (such as soft drinks, filled cookies, snacks, instant soups, and ready-to-heat products); foods need to be physically and financially accessible; eating patterns with sufficient quantity and quality; and derived from sustainable production and distribution practices [8].

However, the transition to healthier and more sustainable eating patterns might negatively impact the cost of diets, especially in LMICs [9]. In addition to the potentially higher cost of healthy diets, these countries face high levels of income inequality, making it difficult for low-income families to have access to adequate food [9]. This relationship can be observed in studies of food price trends carried out in Brazil. Although a previous study in Brazil showed that a diet based on fresh and minimally processed foods would be cheaper than a diet rich in UPFs by 2026 [10], the scenario has changed drastically over the years, where there is an increased in the cost of diets, mainly due to the increase in inflation on natural and minimally processed foods [11–13]. Understanding the distribution of the costs of dietary patterns provides support for the implementation of public policies that promote healthy and sustainable food systems in an accessible way for the population [1, 3].

Given the complexity in the determination of the relative price and affordability of ‘less healthy’ vs. ‘healthy’ foods, meals and diets, the *International Network for Food and Obesity/Non-communicable Diseases Research, Monitoring and Action Support* (INFORMAS) has developed a standardized protocol for these analyses. For the more complete approach involving diets, the use of DIETCOST identifies the cost of a wide variety of healthy and sustainable diets and compares it with the cost of current diets [14, 15], always considering cultural aspects. Thus, the objective of this study was to identify the differential in cost and environmental impact measures between the current diet of the Brazilian population and healthy and sustainable diets.

## Methods

### Study design and population

This modeling study is based on a combination of data from the most recent Brazilian Household Budget Survey (HBS) 2017/18 [16], and the Footprints of Foods and Culinary Preparations Consumed in Brazil database [17].

The national HBS is carried out periodically (every decade) by the Brazilian Institute of Geography and Statistics (IBGE) as a cross-sectional survey, relying on a probabilistic sample of households in the country [16]. We emphasize that although the data is from 2017/2018, this is the most recent source of data for estimating consumption structures, expenses and income of Brazilian families based on the analysis of their family purchases. For the 2017/2018 survey the selected sample consisted of 57,920 households. A detailed description of the sampling process is available elsewhere [16].

The 2017/2018 HBS data of interest for this study is the register of all food and beverage expenses by each household for family consumption and family income. Food and beverage expenses were registered directly in digital format by a family member or IBGE interviewer (when solicited) for a period of seven consecutive days. The interviews were spread over a year to capture the seasonality of food and its costs. Detailed information was available for each record of food and beverage expense data (item description, total quantity acquired in g or ml, and the value expended were used). Expense values were deflated to represent January 15, 2018, the reference date for inflation data from the survey [16]. The total income of each family was calculated by summing all the monetary and nonmonetary income obtained from household members during the one-month period [16]. The currency used in the research is in Brazilian reais (BRL$).

The Footprints of Foods and Culinary Preparations Consumed in Brazil database includes environmental impact parameters for most foods and culinary preparations commonly consumed in Brazil [17]. The database is based on the Life Cycle Analysis (LCA) of products and estimates environmental impact parameters, such as the carbon footprint (CF) and water footprint (WF). CF is based on the total direct or indirect emission of greenhouse gases into the environment during the life cycle of a product. The mass unit (gram, kilogram, or tonne) used is converted into carbon equivalent (CO2eq) based on the polluting potential of each gas. WF is based on the amount of water used for production directly or indirectly, expressed in volume of water (liters) [17]. Information is available for 985 codes of foods and culinary preparations consumed in Brazil and was collected from international databases and scientific sources [17]. The use of environmental impact parameters is based on the amount referring to 1 kg (1000 g) of the edible part of the food. The database was created based on HBS 2008/2009 and has the same correspondence system as HBS (International Convention on the Harmonized Commodity Description and Coding System). A detailed description of the survey and calculation of environmental impact parameters are available in a separate publication [17].

### Data organization

Information was collected for 1800 food items in the HBS (brand information was not collected) [16]. All the data corresponding to the same food were combined (total weight and expense value). Then, very similar items (such as varieties of bananas) were also combined. Items with less significant contributions to total consumption (< 1 g per person per day) were excluded. A list of the most consumed and relevant foods in the population’s diet was subsequently identified (*n= 97)* . The nonedible fraction of each item, such as peels, shavings, and pits, was removed when appropriate, and the list of most consumed items was then linked to data on the nutritional composition of foods [18], allowing characterization of purchases according to their caloric, macronutrient, and micronutrient content. Subsequently, environmental impact indicators were also added for each item (CF, WF) based on LCA information [17].

The unit price (R$/g or R$/ml) was obtained by dividing the total expense value by the quantity purchased (in weight or volume (g or ml)). The total amount of energy (Kcal) acquired, and the costs were divided by seven and by the number of individuals in the population to express daily per capita consumption and daily expenditure values.

Foods were classified according to a classification system based on the Australian Dietary Guidelines [19], into core and discretionary, enabling comparison of results with studies using the same methodology for other countries [20]. Alcoholic beverages and takeaways were excluded, because they presented a nonspecific pattern of consumption within the database used for this study.

### DIETCOST

DIETCOST is a program based on the Python language, enabling the generation of various diet solutions based on a list of foods and beverages and a set of restrictions imposed in the program. It is an open-source tool available on GitHub [21]. More information about the program can be found in previous documents [14, 15, 20].

DIETCOST presents a variety of diets meeting the applied restrictions (using minimum and maximum values established for each item and the other restrictions imposed, based on food groups and nutritional profiles). The number of diets generated depends on the limit of interactions imposed for the estimation of each scenario and on the constraints imposed on the model (the number of constraints is inversely proportional to the number of diets to be generated given a fixed number of interactions). Diets are generated for each family member individually and then grouped, indicating the average values of the diets obtained for each scenario. Each diet generated is independent of the other and refers to fortnightly diets of a standard reference family (composed of a 45-year-old man, a 45-year-old woman, a 7-year-old girl and a 14-year-old boy) [15].

Since HBS data do not provide information on the intrafamilial distribution of food consumption, the data were adjusted to simulate the consumption levels of each member of a household consisting of a 45-year-old man, a 45-year-old woman, a 7-year-old girl and a 14-year-old boy (based on the INFORMAS food price protocol used to carry out analyses in the DIETCOST program) [15]. This procedure consisted, essentially, of adjusting average consumption to the given age group and sex based on an adult-equivalent scale (Supplement S1) [22]. This procedure does not affect the proportion (%) of foods in the total diet of all members or relationship between their amounts.

In the present study, three scenarios were analyzed, the current diet among Brazilians, the BDG diet, and the EAT-Lancet diet. We defined a minimum of 100 diets per scenario for further analysis (between 1 and 2 million interactions). Current family diets were based on the most consumed foods by Brazilians, obtained from the 2017/18 HBS and adjusted to represent a diet with energy content similar to the recommendations used in the BDG and EAT-Lancet diets.

To avoid culturally unacceptable diets for the current diet, with foods outside the standard consumed by the population, we used the lower and upper bounds (5th and 95th percentiles) of the total grams acquired, identified directly from the original distribution for each item (applying a process analogous to that previously described directly to the set of acquisitions of each household). Furthermore, for other diets, using the same foods consumed by the population to maintain the cultural standard of diets.

The BDG and EAT-Lancet diets were based on the dietary recommendations of the BDG [8, 23] and the EAT–Lancet Commission [5], respectively. Since the BDG and the EAT–Lancet Commission do not have recommendations for the consumption of macro -and micronutrients, the recommendations for energy consumption and nutritional restrictions for macronutrients (carbohydrates, free sugars, proteins, total and saturated fat, and total fiber) and micronutrients (sodium) and their minimum and maximum intervals followed an approximate intake pattern of 2,000 kcal/day, based on the World Health Organization (WHO) [24], United States Institute of Medicine (IOM) [25] and international Dietary Reference Intakes (DRI) [26] (Table 1).

**Table 1:** Nutrient targets and food groups of healthy, sustainable, and current diets in Brazil

	Current isocaloric	Brazilian Dietary Guidelines (BDG)	EAT-Lancet
**Energy (kcal/day /person)** ^**a**^	Energy requirements: same as BDG and EAT-Lancet diet	Energy requirements for normal BMI (DRI) ± 3%: G 1587; B 2750; W 2200; M 2900	Energy requirements for normal BMI (DRI) ± 3%: G 1587; B 2750; W 2200; M 2900
**Carbohydrates (%kcal)**	HBS mean: 40–58	WHO range 55–75	WHO range 55–75
**Protein (%kcal)**	HBS mean: 12–18	WHO range: 10–15	WHO range: 10–15
**Total fat (%kcal)**	HBS mean: 26–39	WHO range 15–30	WHO range 15–30
**Saturated fat (%kcal)**	HBS mean: 8–12	WHO range: <10%	WHO range: <10%
**Added sugar (%kcal)**	HBS mean: 6–9	WHO range:<10%	EAT-Lancet report.: <5%
**Fiber (g/1,000 kcal)**	HBS mean: 7–19	IOM min.: 14 g	IOM min.: 14 g
**Sodium (mg/day/person)**	HBS mean: 3352–7259	IOM (UL): G 1900; B 2300; W 2300; M 2300	IOM (AI-UL): G 1900; B 2300; W 2300; M 2300
**Fruit (%kcal)**	HBS mean: 2–5	BDG study: 4–4,5	EAT-Lancet report: 4–7
**Vegetables (%kcal)**	HBS mean: 1-1.5	BDG study: 1–1,5	EAT-Lancet report: 2–4
**Grains and starchy vegetables (%kcal)**	HBS mean: 14–26	BDG study: 26–36	EAT-Lancet report: 23–44
**Legumes and nuts (%kcal)**	HBS mean: 7–12	BDG study:10	EAT-Lancet report: 16–30
**Animal protein sources (%kcal)**	HBS mean:1–7	WHO:0–100	EAT-Lancet report: 4–8
**Red meat (g/day/person)**	HBS mean: 32–47	WHO:0–100	EAT-Lance report t: 0–28
**Dairy (%kcal)**	HBS mean: 2–4	BDG study:4–5	EAT-Lancet report: 4–8
**Discretionary foods (%kcal)**	HBS mean: 9–25	BDG study: 9–18	EAT-Lancet report: 0
**Sauces and spreads (%kcal)**	HBS mean: 1–4	BDG study: 1	EAT-Lancet report: 0
**Sugar-sweetened beverages (%kcal)**	HBS mean: 1–6	BDG study: 1–2	EAT-Lancet report: 0

*Note*: The DIECOST program was used to specify the targets used. DRI: Dietary Reference Intake (Dietary Reference) [26]; BMI: body mass index; WHO: World Health Organization [24]; UL: Tolerable Upper Intake Level [25]. Animal protein sources: Red meat, poultry, seafood, eggs. HBS: Household Budget Survey [16]; BDG study [23]; EAT-Lancet: EAT-Lancet Commission report [5]. G: girl 7 years old; B: boy 14 years old; W: woman 45 years old; M: male 45 years old

The minimums and maximums of each food group followed values identified in a previous study for the BDG diet [23] and the EAT-Lancet diets [5]. Broader nutritional restrictions (± 10% for energy in kcal and ± 30 to 40% for macro and micronutrients) were used for the current diets, enabling a better adjustment of the values obtained for each family member as HBS provides acquisition data and not consumption data. The use of acquired dietary data may be subject to waste and is not directly evaluated at the individual level in the database used. Additionally, although the data were adjusted by family members, the purchase of food is made for the household, and there may be variations in the amounts consumed. Furthermore, the food intakes and their restrictions in the DIETCOST program were constant among family members, with changes only in the nutritional targets and in the amount of food according to the recommendations. In this sense, the composition of each food group was relatively similar between the two diets, with the variation occurring mainly in the amount of each food group and for the current diet (Table 1).

The DIETCOST application [15], manipulated through Visual Studio Code (Python language editor), was used to obtain the diets. Microsoft Excel was used for the elaboration of the database and for the outputs of the diet generated from the DIETCOST program (csv format).

### Statistical analysis

We estimated the means and 95% confidence intervals (95% CI) of the household-level dietary information for nutritional characteristics, food groups, cost, and environmental impact parameters per day. The Kolmogorov-Smirnov tests were performed to verify the normality of the data.

The nutritional composition of the baskets, the cost and the environmental impact of the BDG and EAT-Lancet diets were then compared to the results obtained for the current diet by ANOVA and simple linear regression. ANOVA was used to test differences between the average nutritional characteristics, cost and environmental impact of the diets. The Tukey test was used to correct variables with three or more categories with a p value < 0.05 according to ANOVA. Simple linear regression analyses allowed the assessment of the relationship between each diet scenario (current, BDG, and EAT-Lancet diet) and the environmental impact measures on cost simultaneously regarding statistical significance: 1) environmental impact and cost; (2) environmental impact and nutritional quality (based on each diet scenario, using the current diet as a reference in the model compared to the others); and (3) environmental impact and cost per nutritional quality. For linear regression analyses, we used standardized environmental impact measures (CF and WF), allowing us to interpret of the coefficients in terms of standard deviations.

Stata 16.1 statistical software (Stata Corp., 2019) was used to organize the database for additional statistical analyses.

## Results

**Fig. 1:**
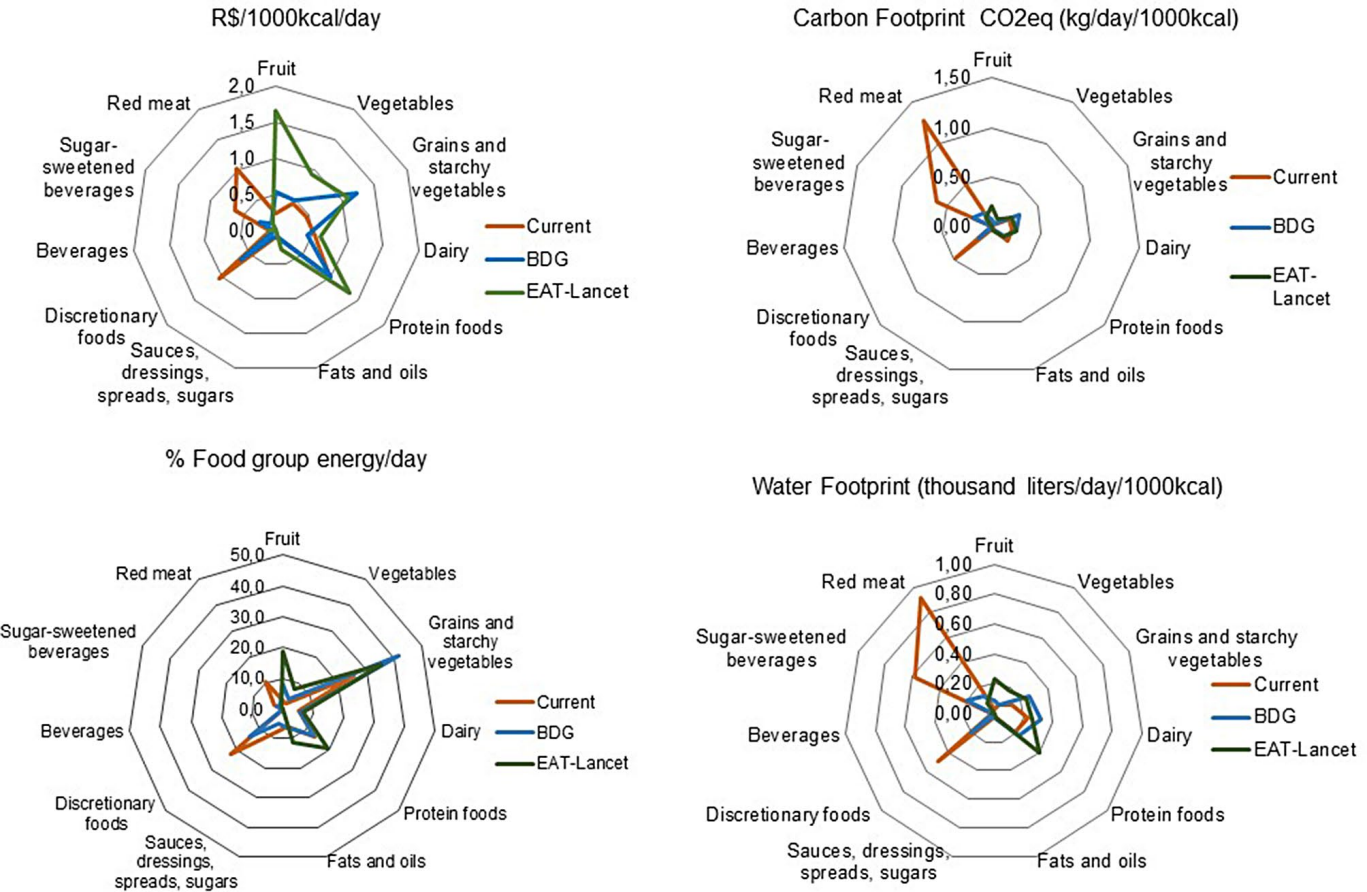
Contribution of food groups to the energy, cost, and environmental impact parameters by 1000 kcal/day. Notes: Protein foods: Poultry, seafood, eggs, legumes, nuts. Beverages: coffee and tea. Current: current diet; BDG: Brazilian Dietary Guidelines diet; EAT-Lancet: EAT-Lancet Commission diet

In total, 4,926 diets were analyzed: 1456 for the current diet, 2756 for the BDG diet and 714 for the EAT-Lancet diet, which adhered to the targets established for nutrients and food groups (Tables 1 and 2). Grains and starchy vegetables (25.4%), discretionary foods (22.5%), protein foods (such as poultry, seafood, eggs, legumes, nuts) (13.8%) and red meat (10.5%) accounted for approximately 70% of the calories in the current diet. The BDG and EAT-Lancet diets had, on average, greater contributions of grains and starchy vegetables (41.2% and 35.2%, respectively), protein foods (13.4% and 19.7%, respectively), fruits (8.1% and 18.6%, respectively), and fats and oils (6.4% and 11.4%, respectively) to the calories of the diet. For these diets, we observed a lower contribution to total calories from discretionary foods (13.9% and 0.0%, respectively), sugar-sweetened beverages (2.1% and 0.0%, respectively), and red meat (1.4% and 0.9%, respectively) when compared to the current diet (Table 3; Fig. 1).

**Table 2:** Cost and nutritional characteristics of diet baskets

	Current isocaloric		Brazilian Dietary Guidelines		EAT-Lancet		*P* value
Number of individual diet baskets	Total: 1456; G:341; B:430; W:139; M:546		Total: 2756; G:659; B:731; W:699; M:667		Total: 714; G:211; B:176; W:169; M:158		
	Mean	95%IC	Mean	95%IC	Mean	95%IC	
Energy (kcal/ day/person)	2437	2406;2468	2386	2367;2405	2233	2195;2271	< 0.001
**Diet characteristics (1000 kcal)**							
Carbohydrates (%kcal)	51.0	50.8;51.3	69.4	69.2;69.5	67.1	66.8;67.4	< 0.001
Protein (%kcal)	18.1	18.0;18.2	12.6	12.6;12.7	13.6	13.5;13.7	< 0.001
Total fat (%kcal)	32.4	32.2;32.6	21.3	21.2;21.5	24.1	23.8;24.3	< 0.001
Saturated fat (%kcal)	12.0	11.9;12.0	7.6	7.5;7.6	9.2	9.1;9.2	< 0.001
Added sugar (%kcal)	9.0	8.9;9.1	4.5	4.4;4.6	0.1	0.1;0.1	< 0.001
Fiber (g/day)	8.0	7.9;8.0	17.0	16.9;17.1	24.6	24.3;24.9	< 0.001
Sodium (mg/day)	1169.2	1151.1;1187.3	733.7	726.8;740.5	432.9	422.9;442.9	< 0.001
Red meat (g/day)	52.5 ^a^	50.5;54.6	7,1 ^b^	6.8;7.4	5.9 ^b^	5.6;6.2	< 0.001
**Cost (1000 kcal)**							
Cost R$/day/person	5.6	5.6;5.7	4.9	4.8;4.9	6.1	6.0;6.1	< 0.001
Cost USD$/day/person	1.8	1.8;1.8	1.5	1.5;1.5	1.9	1.9;1.9	< 0.001
Cost R$/biweekly/person	78.9	78.3;79.5	68.5	68.0;69.1	85.6	84.8;86.4	< 0.001
**Environmental impact parameters (1000 kcal)**							
CF-CO2eq (kg/day/1000 kcal)	3.1	3.0;3.1	1.4	1.4;1.4	1.1	1.0;1.1	< 0.001
WF-(liters/day/1000 kcal)	2,705	2,671;2,739	1,542	1,524;1,561	1,448	1,428;1,469	< 0.001

*Note*: G: girl 7 years old; B: boy 14 years old; W: woman 45 years old; M: male 45 years old. CF: Carbon Footprint; WF: Water Footprint. *P*-value: ANOVA test. Tukey’s correction was applied to those variables with three or more categories with a *P* value < 0.05 according to ANOVA. There was a difference for all groups except for the one indicated with different letters on the same line

**Table 3:** Contribution of food groups to the energy, cost and environmental impact parameters by 1000 kcal/day

	*R*$/1000 kcal/day			% Food group energy/day			Carbon Footprint (kg/day/1000 kcal)			Water Footprint (liters/day/1000 kcal)			
	Current	BDG	EAT-Lancet	Current	BDG	EAT-Lancet	Current	BDG	EAT-Lancet	Current		BDG	EAT-Lancet
**Fruit**	0.24	0.54	1.66	2.9	8.1^¥^	18.6^¥^	0.03	0.07	0.21	30.1		83.3	229.2
95%IC	0.23;0.24	0.53;0.54	1.63;1.69	2.8;2.9	7.9;8.2	18.2;18.9	0.03;0.03	0.07;0.07	0.20;0.21	29.4;30.8		81.1;85.0	224.7;233.7
**Vegetables**	0.45	0.48	0.91	2.3^¥^	3.6^¥^	7.6^¥^	0.03	0.04	0.09	63.4		63.6	185.8
95%IC	0.43;0.46	0.47;0.48	0.89;0.93	2.3;2.4	3.5;3.7	7.4;7.8	0.03;0.03	0.04;0.04	0.09;0.09	61.6;65.2		62.5;64.7	179.4;192.2
**Grains and starchy vegetables**	0.46	1.23	1.09	25.4	41.2^¥^	35.2	0.18	0.29	0.22	136.5		265.4	239.5
95%IC	0.45;0.47	1.21;1.26	1.06;1.12	25.1;25.8	40.8;41.7	34.7;35.7	0.18;0.19	0.29;0.30	0.22;0.23	134.0;138.9		262.0;268.8	234.2;244.9
**Dairy**	0.54	0.44	0.63	5.4^¥^	5.6	6.9	0.20	0.24	0.24	227.5		317.7	261.7
95%IC	0.52;0.56	0.43;0.45	0.61;0.65	5.2;5.5	5.5;5.8	6.6;7.1	0.19;0.20	0.23;0.25	0.23;0.25	220.3;235.8		307.7;327.7	251.2;272.3
**Protein foods**	0.98	1.00	1.34	13.8	13.4	19.7	0.21	0.16	0.16	188.8		223.4	402.2
95%IC	0.96;1.0	0.98;1.0	1.29;1.38	13.5;14.1	13.2;13.5	19.2;20.1	0.20;0.21	0.15;0.16	0.15;0.16	183.2;194.3		219.4;227.5	384.5;419.9
**Fats and oils**	0.11	0.11	0.28	6.1	6.4	11.4	0.02	0.03	0.03	26.1		30.7	35.6
95%IC	0.10;0.11	0.11;0.11	0.26;0.29	5.9;6.2	6.3;6.5	11.1;11.7	0.02;0.03	0.03;0.03	0.03;0.04	25.4;26.8		30.0;31.4	34.4;36.8
**Sauces, dressings, spreads, sugars**	0.11	0.09	0.00	8.0	4.9	0.0	0.02	0.01	0.00	30.0		25.8	0.0
95%IC	0.11;0.12	0.9;0.9	-	7.8;8.2	4.8;5.1	-	0.02;0.02	0.01;0.01	-	29.4;30.7		25.1;26.5	-
**Discretionary foods**	1.04	0.64	0.00	22.5	13.9	0.0	0.48	0.18	0.00	493.1		196.2	0.0
95%IC	1.03;1.07	0.62;0.65	-	22.1;22.9	13.6;14.2	-	0.47;0.50	0.18;0.18	-	482.1;504.1		191.1;201.3	-
**Beverages**	0.08	0.03	0.11	0.0	0.0	0.0	0.01	0.00	0.01	14.3		4.4	19.1
95%IC	0.07;0.08	0.03;0.03	0.11;0.11	0.0;0.0	0.0;0.0	0.0;0.0	0.01;0.01	0.00;0.00	0.01;0.01	13.7;15.0		4.2;4.6	18.3;19.8
**Sugar-sweetened beverages**	0.63	0.25	0.00	3.0	2.1	0.0	0.62	0.21	0.00	581.0		197.9	0.0
95%IC	0.61;0.64	0.24;0.26	-	3.0;3.1	2.0;2.2	-	0.60;0.64	0.20;0.22	-	562.5;599.4		187.1;208.7	-
**Red meat**	1.01	0.11	0.10	10.5	1.4	0.9	1.27	0.16	0.11	914.5		133.9	75.4
95%IC	0.98;1.03	0.10;0.11	0.10;0.10	10.2;10.7	1.3;1.5	0.8;0.9	1.23;1.30	0.16;0.17	0.11;0.012	889.2;939.8		128.1;139.7	70.2;80.5

Notes: Protein foods: Poultry, seafood, eggs, legumes, nuts. Beverages: coffee and tea. Current: current diet; BDG: Brazilian Dietary Guidelines diet; EAT-Lancet: EAT-Lancet Commission diet. Discretionary foods and sugar-sweetened beverages represent ultra-processed foods. ^¥^values ​​outside the limits established in the model

There were significant differences in cost among the diets, with BDG diets being less expensive (BRL$4.9 per day per person/1000 kcal (95% IC: BRL$4.8;4.9)) compared to current (BRL$5.6 (95% IC: BRL$5.6;5.7)) and EAT-Lancet (BRL$6.1 (95% IC: BRL$6.0;6.1)) diets (Table 2). For the current diet, the highest standardized costs per 1000 kcal came from discretionary foods (BRL$1.04 vs. BRL$0.64 for BDG diets) and red meat (BRL$1.01 vs. BRL$0.11 and BRL$0.10 for BDG and EAT-Lancet diets, respectively), while there was a low cost attributed to fruits (BRL$0.24 vs. BRL$0.54 and BRL$1.66 for BDG and EAT-Lancet, respectively) and vegetables (BRL$0.45 vs. BRL$0.48 and BRL$0.91 for BDG and EAT-Lancet diets, respectively) (Table 3; Fig. 1).

Greater environmental impacts were observed for the current diets. The CF per day adjusted for 1000 kcal was 3.1 kg (95% IC: 3.0;3.1) for current diets, 1.4 kg (95% IC: 1.4;1.4) for BDG diets and 1.1 kg (95% IC: 1.0;1.1) for EAT-Lancet diets. For WF per day adjusted to 1000 kcal, consumption was 2,705 L (95% IC: 2,761;2,739) for the current diet, 1,542 L (95%IC: 1,524;1,561) for the BDG diet and 1,448 L (95% IC: 1,428;1,469) for the EAT-Lancet diet (Table 2). Among the food groups, red meat, sweetened beverages, discretionary foods, and dairy were responsible for the major environmental impacts (Table 3; Fig. 1).

A positive association was observed between environmental impact indicators and cost. With every increase of one standard deviation in the standardized CF, there was an associated increase of BRL$0.34 in the daily cost between all diets. Similar increases were observed for the standardized WF (BRL$0.47). Regarding the association between environmental impact and the type of diet, when the current diet was used as a reference, the BDG and EAT-Lancet diets demonstrated lower environmental impact (standardized CF: -1.1; standardized WF: -0.96) (Table 4).

**Table 4:** Regression analyses of cost, environmental impact, and diet

**Environmental impact vs. cost (Model 1)**					
**Cost**	**Coefficient (cost)**	**95% CI**	***P*** **value**	**R** ^**2**^	
Carbon Footprint CO2eq (kg/day) standardized	0.34	0.32;0.36	< 0.001	0.1528	
Water Footprint (liters/day) standardized	0.47	0.45;0.49	< 0.001	0.2340	
**Environmental impact vs. diet (Model 2)**					
**Diet**	**Coefficient (Diet)**	**95% CI**	***P*** **value**	**R** ^**2**^	
Carbon Footprint CO2eq (kg/day) standardized	-1.1	-1.13; -1.06	< 0.001	0.5785	
Water Footprint (liters/day) standardized	-0.91	-0.94; -0.87	< 0.001	0.3925	
**Environmental impact vs. cost per nutritional quality (Model 3)**					
**Current diet**	**Coefficient (cost)**	**95% CI**	***P*** **value**	**R** ^**2**^	
Carbon Footprint CO2eq (kg/day) standardized	0.48	0.42;0.54	< 0.001	0.3019	
Water Footprint (liters/day) standardized	0.58	0.54;0.61	< 0.001	0.3571	
**BDG diet**	**Coefficient (cost)**	**95% CI**	***P*** **value**	**R** ^**2**^	
Carbon Footprint CO2eq (kg/day) standardized	0.20	0.19;0.22	< 0.001	0.2891	
Water Footprint (liters/day) standardized	0.32	0.30;0.34	< 0.001	0.3048	
**EAT-Lancet diet**	**Coefficient (cost)**	**95% CI**	***P*** **value**	**R** ^**2**^	
Carbon Footprint CO2eq (kg/day) standardized	0.07	0.05;0.09	< 0.001	0.0886	
Water Footprint (liters/day) standardized	0.25	0.22;0.28	< 0.001	0.3138	

*Note*: Cost/day/1000 kcal; BDG: Brazilian Dietary Guidelines diet; EAT-Lancet: EAT-Lancet Commission diet. Construction of association models: Model 1: cost was adopted as the outcome and environmental impact as explanatory variable. Model 2: environmental impact was adopted as the outcome and nutritional quality (based on each diet scenario, using diet current as a model reference (Current Diet x BDG Diet x EAT-Lancet)) as explanatory variable. Model 3: cost was adopted as the outcome and environmental impact as explanatory variable per nutritional quality (based on each diet scenario). *P* value: model significance value

The association between the environmental impact and the adjusted cost of the studied diets reinforces the observed relationship between the increased environmental impact and higher cost, particularly among the current diets. An increase of one standard deviation in the standardized CF corresponds to an increase of BRL$0.48 in the cost of the current diet. This pattern holds true for the other environmental impacts studied (standardized WF: BRL$0.56). Although there was a similar trend between environmental impact and cost for the BDG (standardized CF: BRL$0.20; standardized WF: BRL$0.33) and EAT-Lancet (standardized CF: BRL$0.04; standardized WF: BRL$0.18) diets, the impact on diet cost was less pronounced (Table 4).

## Discussion

We analyzed the cost and environmental impact of current, healthy and sustainable diets in the Brazilian population based on a standardized protocol developed by INFORMAS. Our results highlight the significant influence of the nutritional quality of diets on economic and environmental aspects. Notably, we observed that in comparison with current diets, BDG diets exhibited a more favorable cost profile, suggesting that adhering to BDG recommendations can yield financial benefits for the Brazilian population. However, EAT-Lancet diets had slightly higher costs than the current diets. Our study identified a clear association between dietary choices and environmental impact. Current diets were associated with the highest CF and WF. The positive correlation between environmental impact indicators and cost further highlights the interplay between diet, sustainability, and economics, where greater environmental impact was associated with higher costs for all diets, especially those associated with current diets.

Several countries already monitor the costs associated with healthier and more sustainable diets on a regular basis, highlighting the need for interventions that enhance access to nutritious foods while mitigating planetary impact [20, 27–31]. A 2011 study analyzing food price data from 159 countries assessed the economic impact of the EAT-Lancet diet [27]. The average daily cost of the EAT-Lancet diet (2,503 kcal/day) was US$2.84 across all countries, with higher costs in high-income countries (US$2.66) than in low-income countries (US$2.42). Using linear programming, a nutritionally balanced reference diet was found to cost 1.6 times less than the EAT-Lancet diet. The latter was deemed unaffordable for many families, particularly in low-income countries [27]. The cost of healthier diets disproportionately affects lower socioeconomic groups (SEGs) [27].

In a 2011/2012 Australian study, the cost of healthy diets aligned with the Australian Dietary Guidelines was investigated across income quintiles [31]. Overall, the cost of the healthiest diet was lower than the usual diet across all quintiles. For the usual diet there was a significant budget allocation to UPF across quintiles and higher costs of alcoholic beverages in the highest quintile, while healthy foods and beverages had a greater impact on the lowest quintiles [31].

Some studies in middle- and high-income countries, following the INFORMAS protocol, have examined dietary costs [20, 28–30]. In Mexico, both healthy and sustainable diets were cheaper than the current isocaloric diet [28]. Conversely, in New Zealand [20, 29] and Argentina [30], healthy [20, 29, 30] and sustainable [29] diets tended to be more expensive than current diets. In New Zealand, studies have examined scenarios with [20] and without [29] alcoholic beverages and takeaways, both showing higher costs for current diets. In Argentina, although alcoholic beverages were included in current diet calculations, their impact on cost was minor compared to fruits, vegetables, and meats, which also contributed significantly to healthy diets [30].

In our study, the cost finding is mostly explained by the reduction of UPF and red meat in the BDG and EAT-Lancet diets (BDG diets are lower in discretionary foods and red meat in comparison to current diets, while EAT-Lancet diets have no discretionary foods or sweetened beverages and have the lowest amount of red meat). Replacing these groups with fresh vegetables, grains and starchy vegetables imposes an economic benefit in the Brazilian price scenario. Although fruits and vegetables are recognized for their high cost per calorie, grains and pulses tend to be low-cost items per kg, resulting in an overall reduction in cost, a trend akin to findings in other countries [27, 28]. Even though the greater proportion of fruits and vegetables present in the EAT-Lancet diets had an impact on the cost of the diet, we still observed an economic benefit due to the lower amounts of red meat and UPF in this diet.

However, although fresh and minimally processed foods were cheaper than UPF in our price scenario (2017/2018 data), projections suggest that the reverse will be true in 2026 [10] or even sooner, as a deleterious effect of the Covid-19 pandemic [11]. In 2022, due to the effects of the Covid-19 pandemic, the foods that had the biggest impact on food cost were tubers, roots and vegetables, fruits, flour, and pasta, milk, and dairy products [13].

In addition to food costs, our study also assessed the environmental impact of diets. Both the BDG and EAT-Lancet diets, as expected, demonstrated reduced environmental impacts, indicating that aligning dietary choices with health and sustainability goals can yield mutually beneficial outcomes. Moreover, the observed correlation between higher environmental impact and higher dietary costs, which is particularly evident in current diets, underscores it is critical to promote more sustainable eating habits.

Given the approximate cost between current and EAT-Lancet diets, along with the environmentally advantageous aspects of the latter, advocating the adoption of more sustainable diets seems to be a good choice. This parallel is evident in a study conducted in New Zealand, examining diet costs and their connection to GHG emissions [29]. Like our findings in Brazil, this New Zealand study revealed a direct relationship between rising dietary costs and greater climate impacts, particularly within the context of current diets [29]. This relationship holds true within each dietary pattern as well, where an increase in climate impact corresponds to an increase in diet costs [29]. Furthermore, even with the somewhat high cost of the EAT-Lancet diet, which is far from the reality of food consumption in the Brazilian population, we reinforce that choices based on BDG, in addition to presenting a lower cost, have a characteristic closer to the eating habits of the Brazilian population [23], thus being more accessible and making it possible to be a first step toward change.

A study in Brazil using 2008/2009 data employed linear optimization techniques to create culturally acceptable, healthier diets targeted at reducing GHG emissions, comparing them with the cost and sustainability of the current diet (2000 kcal) [32]. The modeled healthier diet showed lower emissions (3.93 kgCO2eq) but higher cost (US$2.67) compared to the current diet (4.40 kgCO2eq; US$2.16) [32]. Adhering to these dietary recommendations would increase overall diet expenditure by 14 to 24%, mainly by promoting higher fruit and vegetable consumption [32]. Another study with 2017/2018 data found that reducing beef and UPF purchases combined could lead to a 21.1% reduction of the CF and a 20.0% reduction in the WF [33].

In this sense, it is imperative to recognize that while BDG and EAT-Lancet diets have reduced environmental impacts, widespread adoption may require targeted policy interventions, educational initiatives, and infrastructural support, reflecting the need for cultural shifts in dietary habits. Especially when we consider the current diet of the Brazilian population, with low consumption of fruits and vegetables, moderate consumption of red meat and dairy and ultra-processed products [33]. Furthermore, Brazil has seen a progressive increase in UPF and animal protein consumption over time [16]. Studies suggest reducing the intake of these products for better health and environmental outcomes [5, 34].

Through a more comprehensive analysis aimed at identifying the effect of environmental impact on the costs of different diet types, we observed that adopting BDG diets can effectively reduce climate impacts while incurring lower costs. Moreover, adhering to recommendations centered on healthier and more sustainable diets, such as the EAT-Lancet guidelines, can be achieved at a cost similar to that of current diets. It is important to underscore that, with the utilization of more current and extensive data regarding food prices in the country, we have noted higher emissions in the environmental impact indicators than what was observed in studies employing earlier data [32, 35]. This value significantly exceeds the recommended estimates for mitigating climate impacts [35], thereby emphasizing the necessity for strategic interventions to rectify this situation.

Reconfiguring health-focused and sustainable food systems requires coordinated policies across sectors such as agriculture, health, education, and the environment. Multisectoral initiatives are crucial at the local, national, and international levels to promote sustainable production and improve planetary and population health [3]. To this end, policies and initiatives aligned with the Sustainable Development Goals (SDGs) outlined in the 2030 Agenda play a pivotal role [36].

Dietary patterns that follow the nutritional guidelines put forth by the WHO [3] and the BDG [8], which advocate for reduced consumption of UPFs and sweetened beverages, along with an increased intake of natural and minimally processed plant-based foods have the potential to decrease the environmental impact of diets [3]. Furthermore, it is necessary to consider clearer recommendations to reduce the consumption of red meat and animal-based products in BDG. Complementary strategies such as minimizing the use of plastics and plastic-derived materials in food packaging, opting for locally sourced and seasonal foods, and reducing food waste, also yield favorable environmental outcomes [3]. Financial incentives, such as subsidies for fresh produce, as well as the imposition of taxes on UPFs, can represent a pertinent economic intervention, fostering the adoption of healthier, sustainable diets and mitigating their associated costs [34]. By intertwining these multidimensional strategies, a comprehensive approach can be developed to guide individuals and societies toward more sustainable and health-conscious dietary patterns.

Limitations should be noted for a better interpretation of our findings. The comparison of the cost of the diets considered only the cost associated with the food items, without considering the costs associated with preparing and obtaining them. The environmental impact on each food was obtained by an average of data from other countries and studies [17], which may vary according to the region and type of food purchase. Another limitation is that the price data used were from 2018, the last food acquisition survey available in the country. Given the changes in food prices observed more recently, especially due to the Covid-19 Pandemic and economic changes in Brazil [11, 13], it is believed that the scenario may have worsened in relation to the higher cost of natural and minimally processed foods, reinforcing the scenario of the differences found between the costs of diets. It should also be noted that the HBS data refer to household purchases, and that an important part of people’s consumption is related to consumption outside the home. However, the use of total nutrients established by international references [24–26] made it possible to analyze the appropriate total calories for each individual. Furthermore, in the application of diet modeling, certain food groups did not meet the established nutrient thresholds. This discrepancy may have impacted the final cost of the diet.

## Conclusions

Our study provides valuable insights into the intricate relationships among dietary choices, costs, and environmental consequences. We observed that the adoption of BDG did not negatively impact costs, while for the EAT-Lancet diet, the impact was slightly greater. However, considering the environmental impact, the CF and WF of the current diets were almost twice those of the BDG and EAT-Lancet diets. In addition, the lowest cost within each diet was related to the lowest environmental impact, especially among the BDG and EAT-Lancet diets. By shedding light on the potential synergies between health, sustainability, and economics, we contribute to the ongoing dialog on shaping more responsible and resilient food systems. In this sense, public policies that aim to encourage the health and sustainability of the Brazilian diet are essential, such as the already implemented warning labeling of processed foods. However, additional efforts such as the implementation of tax incentives for healthy foods concomitantly with the taxation of unhealthy products, as already under discussion in the ongoing tax reform in the country, could promote a healthier, more sustainable, and fairer diet for the Brazilian population.

### Electronic supplementary material

Below is the link to the electronic supplementary material.

Supplementary Material 1

## Data Availability

The dataset supporting the conclusions of this article is available in the IBGE repository [< https://www.ibge.gov.br/estatisticas/sociais/saude/24786-pesquisa-de-orcamentos-familiares-2.html?=&t=microdados>>] and Footprints of foods and culinary preparations consumed in Brazil repository [< https://osf.io/gs4cy/>> ].

## Abbreviations

BDG: Brazilian Dietary Guidelines
CF: Carbon footprint
CO2eq: Carbon equivalent
DRI: Dietary Reference Intakes
EAT: Lancet diet-EAT-Lancet Planetary Diet
GHG: Greenhouse gas
HBS: Brazilian Household Budget Survey
IBGE: Brazilian Institute of Geography and Statistics
INFORMAS: International Network for Food and Obesity/Non-communicable Diseases Research, Monitoring and Action Support
IOM: United States Institute of Medicine
LCA: Life Cycle Analysis
LMICs: Low-and middle-income countries
UPFs: Ultra-processed foods
WF: Water footprint
WHO: World Health Organization
