# Supplementary material for: Differences in the cost and environmental impact between the current diet in Brazil and healthy and sustainable diets: a modeling study
Source: Nutr J. 2024 Jul 9;23:71. doi: 10.1186/s12937-024-00973-x (PMC11234630; doi:10.1186/s12937-024-00973-x)
Supplement: Supplementary file 1 — Supplementary Material 1 [file 12937_2024_973_MOESM1_ESM.docx]

**Supplement**

Supplement 1: Projection of the number of equivalent adults by estimation of conversion factors in equivalent adults for estimated caloric needs according to age and sex.

| **Age groups** | **Population projection 2018^¥^** | **Equivalent Adult Conversion Factor *** | **Equivalent adult conversion** | |
| --- | --- | --- | --- | --- |
| **Newborns** | |  | |  |
| 0 to <1 | 2,978,233 | 0.29 | 863,688 | |
| **Children** |  |  |  | |
| 1 to 3 | 8,867,360 | 0.51 | 4,522,354 | |
| 4 to 6 | 8,754,136 | 0.71 | 6,215,437 | |
| 7 to 10 | 11680,287 | 0.78 | 9,110,624 | |
| **Men** |  |  |  | |
| 11 to 14 | 6,242,278 | 0.98 | 6,117,432 | |
| 15 to 18 | 6,613,226 | 1.18 | 7,803,607 | |
| 19 to 24 | 10,467,326 | 1.14 | 11,932,752 | |
| 25 to 50 | 40,063,092 | 1.14 | 45,671,925 | |
| 51 and more | 22,077,899 | 0.9 | 19,870,109 | |
| **Women** |  |  |  | |
| 11 to 14 | 5,985,116 | 0.86 | 5,147,200 | |
| 15 to 18 | 6,377,510 | 0.86 | 5,484,659 | |
| 19 to 24 | 10,276,564 | 0.86 | 8,837,845 | |
| 25 to 50 | 41,685,856 | 0.86 | 35,849,836 | |
| 51 and more | 26,426,017 | 0.75 | 19,819,513 | |
| **Total** | 208,494,900 | - | 187,246,979 | |

¥ Census projections for the year 2018.Reference: Instituto Brasileiro de Geografia E Estatística. Projeções da População. Rio de Janeiro: IBGE. 2020. https://www.ibge.gov.br/estatisticas/sociais/populacao/9109-projecao-da-populacao.html?=&t=resultados . Accessed 12 Jan 2023.

* Estimates of consumption in kcal for equivalent adults. Reference: Claro RM, Levy RB, Bandoni DH, Mondini L. Per capita versus adult-equivalent estimates of calorie availability. Cad Saude Publica. 2010 Nov;26(11):2188-95.
